# Is There a Risk of Yellow Fever Virus Transmission in South Asian Countries with Hyperendemic Dengue?

**DOI:** 10.1155/2013/905043

**Published:** 2013-12-03

**Authors:** Suneth B. Agampodi, Kolitha Wickramage

**Affiliations:** ^1^Department of Community Medicine, Faculty of Medicine and Allied Sciences, Rajarata University of Sri Lanka, Saliyapura, Sri Lanka; ^2^Tropical Disease Research Unit, Faculty of Medicine and Allied Sciences, Rajarata University of Sri Lanka, Saliyapura, Sri Lanka; ^3^Health Department, International Organization for Migration (IOM), Colombo, Sri Lanka

## Abstract

The fact that yellow fever (YF) has never occurred in Asia remains an “unsolved mystery” in global health. Most countries in Asia with high *Aedes aegypti* mosquito density are considered “receptive” for YF transmission. Recently, health officials in Sri Lanka issued a public health alert on the potential spread of YF from a migrant group from West Africa. We performed an extensive review of literature pertaining to the risk of YF in Sri Lanka/South Asian region to understand the probability of actual risk and assist health authorities to form evidence informed public health policies/practices. Published data from epidemiological, historical, biological, molecular, and mathematical models were harnessed to assess the risk of YF in Asia. Using this data we examine a number of theories proposed to explain lack of YF in Asia. Considering the evidence available, we conclude that the probable risk of local transmission of YF is extremely low in Sri Lanka and for other South Asian countries despite a high *Aedes aegypti* density and associated dengue burden. This does not however exclude the future possibility of transmission in Asia, especially considering the rapid influx travelers from endemic areas, as we report, arriving in Sri Lanka.

## 1. Background 

In February 2012, mainstream media reported that Sri Lanka faced a “threat” of local transmission of yellow fever (YF) due to the repatriation of clusters of Sri Lankans from West African countries where the disease was endemic [1]. Since January 2012, large numbers of Sri Lankans were intercepted as they tried to migrate to Canada through “irregular” means (via human smuggling operations). This incident was communicated to the media by a health official as a threat of YF transmission in Sri Lanka creating a major public health panic [2]. Sri Lanka is hyperendemic to dengue with the dengue virus causing 220 deaths and 44,855 cases in 2012 alone [3]. The transmission of dengue in Sri Lanka is mainly due to the vector mosquito *Aedes aegypti*, which is also the competent vector for YF. Since the mosquito vector *Aedes* is abundant in Sri Lanka, it appeared logical to conclude that Sri Lanka is a high risk country for YF transmission. The epidemiological unit of the Ministry of Health in Sri Lanka formally alerted the public health system of this risk [4].

However, an evidence-based public health practice requires rigorous synthesis of available scientific evidence to move beyond a singular plausible explanation [5]. We performed an extensive review of literature pertaining to the risk of YF transmission in the South Asian region, in order to understand the probability of actual risk and to assist evidence informed public health policies.

## 2. Disease History and Epidemiology

YF is viral hemorrhagic fever caused by the yellow fever virus, prototype member of the genus *Flavivirus* in the family Flaviviridae. It has a single serotype and five genotypes. The virus is transmitted by vector mosquito primarily by *Aedes* spp. in Africa and *Haemagogus* spp in South America. There are three epidemiologically different infectious cycles in which the YF virus is transmitted from mosquitoes to humans or other primates. In the sylvatic “Jungle” cycle, monkeys act as host and *A. africans* and other *Aedes* spp as the vector. In the savanna (intermediate) cycle, noted only in Africa, monkeys and humans act as hosts with *Aedes* spp as vector. Finally, in the “Urban” cycle only *Ae. aegypti* is involved with human as hosts. *Ae. aegypti* mosquito is well adapted to urban centres and can also transmit other diseases such as dengue and chikungunya.

The spectrum of the clinical disease may vary from mild flu like disease to classical triphasic hemorrhagic fever with hepatorenal involvement. Only around 15–25% of the cases progress to the period of intoxication and 20–50% of patients with end organ impairments die [6]. Before the development of YF vaccine, YF was one of the most feared death specially in the Atlantic trade route, which was known as “Yellow Jack” and also the basis for the legend “Flying Dutchman” [7]. The first documented outbreak of YF was reported from Guadeloupe and Yukatan in 1648 [8]. Though the disease originated from West African countries, devastating epidemics of YF were reported from tropical and subtropical Americas in the 18th and 19th centuries. It then spread to European countries through travel and trade routes, causing epidemics in France, Spain, England, and Italy [9]. A resurgence of the disease occurred in late 1920's and early 1930's due to heavy outmigration of nonimmune European populations to endemic countries and through trade routes [10].

The successful introduction of the YF vaccine and mass immunization campaigns in West Africa in 1940's lead to a significant reduction of disease in high endemic countries. The largest recorded outbreak in the post-YF vaccine era occurred in Ethiopia during 1960–1962, with more than 100,000 people in the Omo and Didessa river valleys acquiring YF leading to 30,000 deaths [12]. Even though YF reemerged as a priority global agenda since this outbreak, it continued to cause epidemics in endemic countries, also spreading to West African countries where cases were never previously reported and to the Eastern Mediterranean region [7].

**Figure 1:**
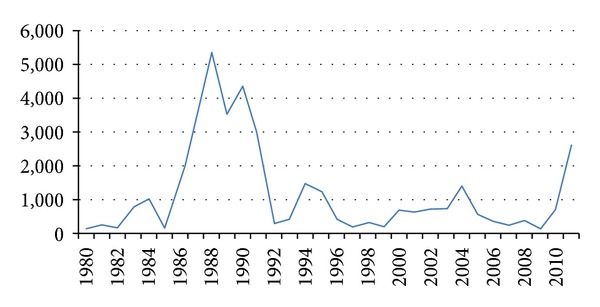
WHO surveillance data on reported cases of yellow fever 1980–2011.

Of importance is the complete absence of yellow fever in South Asia before the introduction of the vaccine. The World Health Organization (WHO) YF surveillance database from 1981 to 2011 showed 42 countries reported YF during the last 30 years, with major outbreaks in 1987–1991 period (Figure 1). However, WHO estimates an annual caseload of 200,000 cases with 30,000 deaths due to underreporting. The “at risk” population is estimated at 900 million people living across 45 endemic countries (32 African and 13 Latin American). The revised global YF risk map in 2011 classified 27 of 32 endemic countries in Africa as having risk for YF transmission and five countries as having “low potential” for exposure to YF (Table 1) [11].

Despite the possibility of the spread of YF from East Africa to Asia being hypothesized as early as 1934 [13], YF has never been reported in Asia. WHO also cautions the “potential for outbreaks” to occur in Asia [14], especially in the context of growing migration flows and increasing *Aedes* mosquito densities across many countries such as India [13]. Different theories have presented to explain this “mystery.” These are explored with available evidence and in relation to Sri Lanka.

## 3. Mapping Theories and Evidence Base

### 3.1. Theory That YF Was Never Introduced to Asia

The first theory postulates that YF has never been introduced to Asia. Some investigators have argued that the absence in Asia could be due to failed introduction of YF in Asia prior to the modern transportation era [14]. However, during the 17th century, world trade and travel by Europeans involved mainly African and Asian nations. Though the majority of slave trades were not routed in the direction of Asia, the European nations involved in such trades that concurred in West Africa also travelled to Asia. The “Coolie trade” in the 18th and 19th centuries involved the migration flow of Indian and Chinese labourers towards African, Latin American, and Caribbean countries, where YF was endemic. This trade which opened both inbound and outbound human migration flows provided ample opportunities for the introduction of YF to Asia.

In the 20th century, world travel increased in exponential proportions. Further opportunities for the introduction and spread of YF to Asia from South America are linked to the opening of the Panama Canal in 1914, which brought Asiatic ports into contact with those in South America where YF is endemic [15, 16].

This argument may be contested in light of evidence that YF had spread to Latin America, become endemic, and resulted in outbreaks in North America and Europe even before the air travel has been invented. Spread of yellow fever from Africa to America was due to slave trade and the first documented outbreak outside Africa was reported from Yukatan in 1648 [9]. Spread of yellow fever to Europe was through sea ports and all initial outbreaks were reported from Spanish and Portuguese ports [17]. Spice trade in South and South East Asia was started as early as in 1498 by Portuguese and they controlled almost all sea ports of India (since 1498), Sri Lanka (since 1597), Maldives (since 1518), Malacca (since 1511), and several other countries over a century and half. During the same period they were extensively involved in slave trade in YF endemic African countries [18]. Subsequent colonial emperors in Asia (Dutch and English) also had large YF outbreaks in their own countries (specially in sea ports) during the 18th and 19th centuries [17] but Asia has not been affected. Further, restricted air travel was true for the African region while it was not so for central and Latin American regions where the YF was endemic.

**Figure 2:**
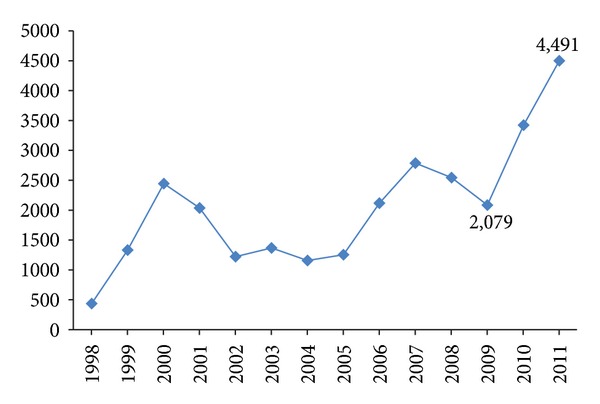
Number of Sri Lankans travelling to yellow fever endemic countries based on registries at Port Health Medical Offices (1998 to 2011).

At present, countries in Asia have a combined population of more than 4 billion persons. Travel statistics are not available for proper estimation of travel dynamics between Asian and YF endemic countries. However, India reported more than 450,000 inbound travellers from Africa and central/south America in 2008, out of which more than 200,000 are estimated to be from countries with risk of YF virus transmission [19]. In Sri Lanka, during the 2007 to 2008 period, the total inbound migration from YF endemic regions was 12,542. The outbound migration from Sri Lanka to YF endemic countries increased rapidly from the end of civil conflict in 2009 (Figure 2), with travellers to Africa, South America, and Middle East comprising 97%, 2%, and 1%, respectively.

The lack of air travel from remote disease endemic areas was a strong alternative explanation to support the theory that YF was never introduced to Asia [6]. It is noteworthy that yellow fever never appeared in Asia even before the discovery of the YF vaccine by Max Theiler in 1937 [20]. Almost all countries in Asia require people travelling to and from YF endemic zones to undertake and/or produce YF vaccination records at ports of entry [21]. Although data for YF vaccination records in Asia are scarce, the literature revealed that 25% of the passengers travelling to Kolkata, India, during the 1982–1984 period possessed valid YF vaccination certificates [22]. As noted in introduction, irregular migration routes such as those stemming from human smuggling and trafficking, before the prevaccination era, provided multiple opportunities for introducing YF to the Asian continent via sea, land, and air routes.

Beyond human hosts, mosquito vectors infected with YF virus may also bring the disease through aircraft or ships. Worldwide distribution of *Culex quinquefasciatus, Aedes aegypti, Aedes albopictus*, and *Anopheles gambiae* and several other mosquito species carried in ships, sailboats, and steamboats, which resulted in the spread of dengue, malaria, and yellow fever, has been well documented in medical literature [23]. Even in the modern world, countries with the highest levels of biosecurity have failed to stop introduction of exotic mosquitoes entering their countries [24]. The theory of failed introduction via migration routes is therefore weak in the context of growing migration flows, growing *Aedes* populations and zones of infestation, and around 200,000 annual cases of YF in endemic countries.

### 3.2. Protective Immunity from Dengue and Other *Flavivirus* Cross-Reactive Antibodies

Asia is considered to be a YF “receptive” area due to the abundance of the competent epidemic vector for urban YF, *Aedes aegypti* mosquitoes. Throughout Asia, especially in South Asian region, this vector is responsible for hyperendemic dengue. In Sri Lanka, the annual case number consistently exceeded 35,000 during last three years, showing a sustained epidemic of dengue fever. Reported seroprevalence of *flavivirus* infection among Sri Lankan children ranged from 34% to 51.4% [25–27]. However, the reported seroprevalence among children less than 11 years had risen to 51.4% in 2013. At the age of 11 years, the prevalence was 71.7%. Seroprevalence studies in India showed that a prevalence of dengue antibodies among adults population is as high as 100% [28].

A hypotheses for the lack of YF in Asia is due to protective immunity conferred from dengue and other *flavivirus* cross-reactive antibodies in populations due to the “original antigenic sin theory” first described by Thomas Francis in 1960 [29]. Cross-reactivity of *flavivirus* antibodies, antigenic properties responsible for this immunogenic property of *flaviviruses,* has been studied extensively [30–35]. During reinfection of dengue due to different serotypes, dengue responsive CD8+ T cells showed low affinity for the infecting serotype and higher affinity for other, probably previously encountered strains [36]. These studies lead to identification of epitopes recognized by dengue serotype-cross-reactive and *flavivirus*-cross-reactive CD4+ CTL [37]. Cross-reactivity of *flavivirus* antibodies created problems in diagnosis of dengue and YF infections [33]. Another study done among Malay soldiers showed that most of them were having antibodies that cross-reacted with YF assay [38]. Experimental hamster models confirmed that the prior heterologous *flavivirus* infection including dengue could prevent fatal YF [39]. When challenged with YF virus, dengue-immune rhesus monkeys showed low viraemia compared to nonimmune monkeys under experimental settings [40]. A single study showed that previous exposure to dengue infection may not prevent yellow fever infection, though it induces an anamnestic immune response. Nevertheless, the study concluded that the severity of the disease could greatly be reduced [41].

Monath argued that dengue immunity could protect against clinical progression of YF infection by reducing viraemia and decreasing the possibility of secondary spread [42]. Historical reports and observational studies have provided supportive evidence for cross-reactivity of dengue and YF antibodies conferring relative protection for those from high dengue endemic areas. As summarized by Vainio and Cutts [7], during the YF epidemics in America in the 19th century, Indian labourers and British troops that served in India were less susceptible for YF [43]. So acute was this observation/realization amongst military leaders that during Napoleonic wars, it was suggested that troops be “seasoned in India” before they were dispatched to West Indies [44]. Further, Indian workers brought to sugar plantations in West Indies were minimally affected during the YF epidemics [45].

Based on a range of historical, experimental, and observational studies and epidemiological data, it appears that previous exposure to dengue and other *flavivirus* provide a compelling hypothesis on the absence of yellow fever in Asia.

### 3.3. Coexistence of Yellow Fever and Dengue Virus in West Africa and South America

Even though the protective immunity theory may partially explain the absence of YF in Asia, the dengue virus has been shown to continually occur in parts of Africa [46] and South America [47]. A challenge and unresolved mystery for scientists propagating the protective immunity hypothesis have been the failure to conclusively explain why dengue and (urban) yellow fever coexist in West Africa.

One explanation for this coexistence is known as the “African hypothesis” and relates to *Ae. albopictus*, an epidemic vector for dengue [48], but with limited capacity for YF transmission [49]. Using a complex mathematical model, Amaku and colleagues showed that the low prevalence of the oriental mosquito *Ae. albopictus* in Africa, combined with a high density of *Ae. aegypti*, could be an alternative explanation for this observation. This simulation model was based on the assumptions that the vector competence of *Ae. albopictus* had shown limited potential to transmit YF [50], that *Ae. albopictus* competes with *Ae. aegypti* [51] with studies documenting a competitive reduction of *Ae. aegypti* by invasive *Ae. albopictus* [52], and that individuals who have recovered from dengue are partially immune to yellow fever. In their model they explained that if the cross-immunity is less than 93% in Africa, then dengue and urban YF could indeed coexist.

### 3.4. Vectorial Capacity

The ability of a mosquito species such as *Ae. aegypti* to serve as a disease vector is determined by its vectorial capacity [53]. Vectorial capacity is influenced by the density, longevity, and competence of the vector including associated environmental, behavioural, cellular, and biochemical factors that influence its association between virus type and host [54, 55]. Vector competence, is a subcomponent of vectorial capacity and is defined by genetic factors that influence the ability of a vector to transmit a pathogen and the inherent tolerance of the vector to ensure viral transmission, infection, and replication [55–57].

Reviews have described an interplay of factors such as mosquito morphology, viral genetics, and environment that govern the transmission of *Flaviviruses* in the *Ae. aegypti* vector [58]. *Ae. aegypti* has two distinct genetic clusters. The first cluster, domestic, and forest populations of *Ae. aegypti* in Africa are included within an ancestral form. The second genetic cluster contains all domestic populations outside Africa. Interestingly, all domestic forms could be assigned back to the human population which they are associated with [59]. Evolutionary aspect of *flavivirus* shows that YF virus as the prototype form with slower evolutionary dynamics compared to other *flaviviruses*, specially to dengue [60]. These two evolutionary pathways of vector and virus could have overlapped and the observed variation of vectorial competencies in harbouring different *flavivirus* could be a part of the evolutionary process. Polymorphism in the vector competence of *Aedes* mosquitoes in disease transmission that occur among geographical samples is largely attributed to such evolutionally pathways [61].

The role of vector competence has also been studied in relation to *flaviviruses* and *Ae. aegypti* [58]. *Flaviviruses*, such as yellow fever, dengue, and West Nile virus differ not only in their interactions with the *Ae. aegypti* mosquito, but also in interactions within viral genotypes [62]. Dengue virus genotypes of Southeast Asian origin have been significantly associated with higher virulence and transmission compared to those from other regions [63, 64]. *Ae. aegypti* is the primary vector for transmission of dengue in Asia which is considered as a possible vector for YF if it ever occurred in Asia. A worldwide genetic variation study of *Ae. aegypti* using 34 mosquito populations showed clearly distinct two major groups of *Ae. aegypti* in Africa and America. Genetic variations of Asian strains were significantly lower compared to African and American strains, which were attributed to historical absences of YF in Asia [65]. Oral susceptibility studies using large number of mosquito populations confirmed the genetic variation of *Ae. aegypti* in YF transmission [66]. Few studies showed genetic foci as well as nongenetic factors in different mosquito populations that determine the susceptibility of *Ae. aegypti* to YF virus [67]. This was further studied and colonization was also shown to have an effect on vector competency through genetic and phenotypic variations [68] which is largely geographically determined. Asian strain was shown to have significantly low competency of YF transmission compared to African and American counterparts in some other studies. Studies done within the African continent also show varying vectorial competencies. As an example, South African strains *Ae. Aegypti* were shown as potentially poor vector of YF [69]. Even in high endemic African countries, some strains of *Ae. aegypti* were shown to be less efficient in transmitting YF virus [70]. Noteworthy is the fact that a few laboratory experiments have shown the Asian strains of *Ae. aegypti* as having the highest infection rates and oral susceptibility to YF [71]. However, YF epidemics such as the 1987 epidemic in Africa, in particular Nigeria, have also been shown to occur with relatively incompetent vector strains, where vector was relatively resistant to infection and transmitted the virus inefficiently [72]. Gubler also reported that Asian vectors could acquire and transmit yellow fever virus [73].

Though some of these molecular evidence and laboratory experiments providing evidence to suggest that vectorial competence may be an alternative explanation for lack of YF in Asia, some studies showed definitive evidence that Asian vectors could acquire and transmit the disease. Thus, this theory is not a strong explanation of absence of YF in Asia.

### 3.5. Genetically Determined Immunity against YF Virus

A large body of evidence, mostly based on laboratory studies and animal models, shows greater range of genetic variation of *flavivirus* infections and genetic determinants [74–77]. In mouse models, innate resistance to *flavivirus* was experimentally shown due to variation in cluster of genes on chromosome 5 and the investigators speculated a possible role for OAS1 in human susceptibility to flavivirusviral infections [78]. Recent studies on dengue have clearly shown genetic determinants of DENV susceptibility, including human leukocyte antigens, blood type, and single nucleotide polymorphisms in immune response genes [79, 80]. Human predisposition to Tick-borne encephalitis virus (another *flavivirus*) was also shown to be associated with SNPs [81, 82]. Though laboratory evidence may indicate a possible genetic determination of yellow fever infection and susceptibility, there is no clear evidence to suggest that the lack of disease in Asian continent is due to human genetic factors. Epidemiological as well as genetic studies targeting this specific objective are needed to confirm the hypothesis.

### 3.6. Viral Interference: Competition of YFV and DENV within Mosquito Cell

Recent in-vitro studies suggest that DENV interferes with the YF virus replication within the mosquito cells, especially where there is a competition between two *flavivirus*. Highly adaptive and evolutionary more advanced, dengue viruses were shown to “win” this competition [83–85]. While no report of dengue and YF coinfection in human beings has been reported hitherto, results from in-vitro studies showing the presence of viral interference may add to the hypothesis of the dominant role DENV serotypes play in the Asian context. One argument against this in-vitro studies is that even during epidemics DENV infected vectors are around 20% [16].

### 3.7. Competitive Exclusion Principle

Combining the evidence from cross-immunity and viral interference within mosquito cells, a generalization of previously suggested competitive expulsion principal [86–88] has also been suggested to explain the absence of YF in Asia. The competitive exclusion principle represents an extreme idealized situation in which only one disease prevails [49]. The principal assumes that mosquitoes and/or humans can be infected by dengue or yellow fever but not by both. Each infection serves as a perfect vaccine for the other infection in both human hosts and mosquito vectors. Based on the evidence described, this exclusion should always favour dengue within hyperendemic Asian countries.

### 3.8. Evidence from Mathematical Modeling

Beyond basic and applied research on YF, mathematical modelling has also been utilized in explaining the mystery of YF in Asia. Amaku and colleges tested several hypothesis in their differential equation model which included the following assumption: Asian *Ae. aegypti* is relatively incompetent to transmit yellow fever; competition between dengue and yellow fever viruses existing within the mosquitoes; when an *Ae. aegypti* mosquito is infected by yellow fever and then acquires dengue, it becomes latent for dengue due to internal competition within the mosquito between the two viruses; cross-immunity between yellow fever and dengue leads to diminished susceptibility to yellow fever in dengue epidemic regions [49]. The model showed an additive effect from all four hypothesis, but the predominant contributing effect was from the cross-immunity hypothesis [88]. A limitation of the model was that it did not consider the genetic susceptibility theory.

## 4. Conclusion 

The probability of “yellow fever never introduced to Asia” and related explanation of geographical barriers are highly unlikely to explain the mystery of YF. Considering other theories we conclude that the probability of risk of local transmission of YF is extremely low in Sri Lanka where dengue is hyperendemic. This does not however exclude the possibility of importation and autochthonous transmission due to factors such as rapidly increasing migrant flows, vector habitat expansion with the forging of new sylvatic territories through climate change, and disrupted or poor vaccination coverage. The H1N1 pandemic proved that despite enhanced surveillance, disease control activities, and travel restrictions, there were many failures in the public health community failing in containing the outbreak.

The current epidemiology shows dengue is mainly transmitted by urban mosquitoes *Ae. aegypti *and *Ae. albopictus*, whilst YF circulates in Africa within predominantly rural areas and mainly within sylvatic mosquitoes. Based on such epidemiological data and those historical, experimental, mathermatical modelling and observational studies described here, provide a compelling argument for the absence of yellow fever in Asia.

Despite what has been described by both media and health administrators as a “conducive” and “enabling environment” for YF transmission in Sri Lanka with rapid population movements from endemic countries and an abundance of the *Ae. aegypti *vector, no evidence of YF transmission has ever been described. Public health awareness and risk communication form a vital function of any health authority. We recommend the use of evidence-based public health approaches rather than a reliance of “simple logic” in determining disease transmission risk. The use of evidence should be a prerequisite in formulating public health announcements and averting potential panic or fear psychosis within general public on autochthonous transmission and outbreaks. A focus on strategies such as ensuring that outbound travellers receive YF vaccination upon receipt of their travel itinerary at least ten days prior to departure and the active surveillance at ports of entry are required. Such approaches have been effective in malaria elimination activities in Sri Lanka [89].

## Figures and Tables

**Table 1:** Classification of countries with risk of yellow fever transmission [11].

Africa		
Countries with risk of yellow fever virus transmission		

Angola	Equatorial Guinea	Mauritania^1^
Benin	Ethiopia^1^	Niger^1^
Burundi	Gabon	Nigeria
Cameroon	The Gambia	Rwanda
Central African Republic	Ghana	Senegal
Chad^1^	Guinea	Sierra Leone
Congo, Republic of the	Guinea-Bissau	Sudan^1^
Côte d'Ivoire	Kenya	Togo
Democratic Republic of the Congo	Liberia	Uganda
Congo^1^	Mali^1^	

Countries with low potential for exposure to yellow fever virus		
Eritrea^1^	Zambia^1^	Tanzania
São Tomé	Somalia^1^	

South America (countries with risk of yellow fever virus (YFV) transmission)		
Argentina^1^	Suriname	Panama^1^
Bolivia^1^	Trinidad and Tobago^1^	Paraguay
Brazil^1^	Venezuela	Peru^1^
Colombia^1^	French Guiana	
Ecuador^1^	Guyana	

^1^These countries are not holoendemic (only a portion of the country has risk of yellow fever transmission).
